# Editing of a Specific Strain of Escherichia coli in the Mouse Gut Using Native Phages

**DOI:** 10.1128/spectrum.01804-22

**Published:** 2022-10-27

**Authors:** Li Ping, Li Zhuoya, Jia Pei, Chen Jingchao, Li Yi, Liu Guosheng, Wang Hailei

**Affiliations:** a College of Life Sciences, Henan Normal University, Xinxiang, China; b Advanced Environmental Biotechnology Center, Nanyang Technological University, Singapore, Singapore; Forschungszentrum Jülich GmbH

**Keywords:** microbial editing, gut microbiome, *Escherichia coli*, phage, host specificity

## Abstract

There is a lack of methodological investigation of the *in situ* functions of bacterial species in microecosystems. Here, we used native phages as a microbial editing tool for eliminating Escherichia coli strain MG1655 labeled with green fluorescent protein (GFP) in the mouse gut. The virulent phages (W1 and W3) possessed host specificity at both the genus and species levels, resulting in an 8.8-log_10_ difference in the titer of viable bacteria after 12 h of phage treatment compared with that in the phage-free control in an *in vitro* test. *In vivo*, they reduced strain MG1655 colonizing the mouse gut at concentrations of 10^6^ to 10^8^ CFU g^−1^ to a 10^2^ CFU g^−1^ level, which is almost undetectable by the plate colony-counting method. Moreover, the impact of phage treatment on the microbial community structure of the mouse gut was not significant (*P* > 0.05), indicating that native phages can effectively edit a target bacterium, with limited perturbation of microbial diversity and relative abundance. Therefore, we developed an engineering technique for investigation of the functions of a specific bacterium by depleting its abundance in microecosystems.

**IMPORTANCE** This report describes a gut engineering technique for investigation of the functions of a specific bacterium. Native phages with host specificity can knock down the corresponding E. coli strain in the mouse gut with limited perturbation of microbial diversity and relative abundance, indicating that they, as a microbial editing tool, can effectively edit the abundance of a target bacterium. Such an approach is undoubtedly of interest in the context of lack of knowledge of how to methodologically study the *in situ* function of a specific species in a complex microecosystem.

## INTRODUCTION

The microbiome, defined as the totality of microorganisms and their collective genetic materials in a well-recognized ecosystem, has become a current focus of scientists and governments. It contains information including the composition and structure of the microbiome, genetic and physiological functions, and even interactions between microbes and their hosts or environments (1–3). The human microbiome, including the gastrointestinal microbiome, respiratory microbiome, reproductive tract microbiome, oral microbiome, and skin microbiome, is closely related to human health, as an integral part of the human body (4). The gut flora has particularly important roles in the nutritional, immunological, and physiologic processes of the host. The human gut harbors at least 100 trillion microbial cells, and the quantity is greater than that of the human body’s own cells (5). Approximately 1,000 to 1,150 species of bacteria colonize the human intestine, with an average of approximately 160 dominant species in each body. The composition of the gut flora has been correlated with intestinal inflammatory bowel disease, diabetes, asthma, liver disease, obesity, mental illness, and cancer, making it an important metabolic organ (6–12).

There is currently a lack of knowledge of how to methodologically study the *in situ* function of a specific species in a complex ecosystem. Generally, knowledge about microorganisms obtained by pure cultivation cannot accurately deduce the role they play in the ecosystem. Current sequencing-based analyses of the microbiota, including metagenomic and 16S rRNA gene sequencing, only permit evaluation of the correlation between a species, genus, or core microbiota and the phenotype of the host by determining the variation in microbial abundance and diversity (13–15). Thus, these relative approaches are limited in revealing the interplay between the specific microorganism or microbiota constituent and host phenotypes or health (16, 17). Various gnotobiotic and germfree (GF) animals, including zebrafish and mice, are also used to study the function of the gut flora (18, 19). However, these animals can only be used to investigate the function of the specific microorganism or microbiota under a simple background setting, and how a species works in a complicated community cannot be uncovered in this manner. This methodological deficiency is a major bottleneck for studies of the function of a specific microorganism in the microbiota, although a few novel techniques are emerging, including isotope probes and quantitative microbiome profiling (17, 20). Theoretically, similar to gene function revealed using gene knockout and complementation techniques, the function of a specific microorganism in a microbiota should be illuminated by microbial knockout and complementation. In fact, the identification of pathogenic bacteria as the causative agents in infectious diseases by Koch’s law follows the principle of microbial complementation, since it is technically easy and operable for a cultivable microorganism. Conversely, strategies to knock out a microbial species in a complicated microecosystem and ensure that its neighbors are not mistakenly removed due to off-target effects are substantial technical challenges.

Bacteriophages (phages), the viruses that infect bacteria, exist widely in the environment, and they have been applied in the prevention, treatment, and control of pathogens or contamination in humans, animals, crops, and foods (21–26). Past research was largely focused on treating ongoing infections using exogenous phages as antibacterial agents. Herein, we used isolated native phages with high host specificity to edit a specific strain of Escherichia coli in the mouse gut. The aim of this work was to evaluate the feasibility of phages as a bacterial editing tool for the gut microbiota and develop an efficient engineering technique for investigation of the functions of a bacterial species by depleting its abundance in microecosystems.

## RESULTS

### Morphology and sequencing analysis of phages.

**FIG 1:**
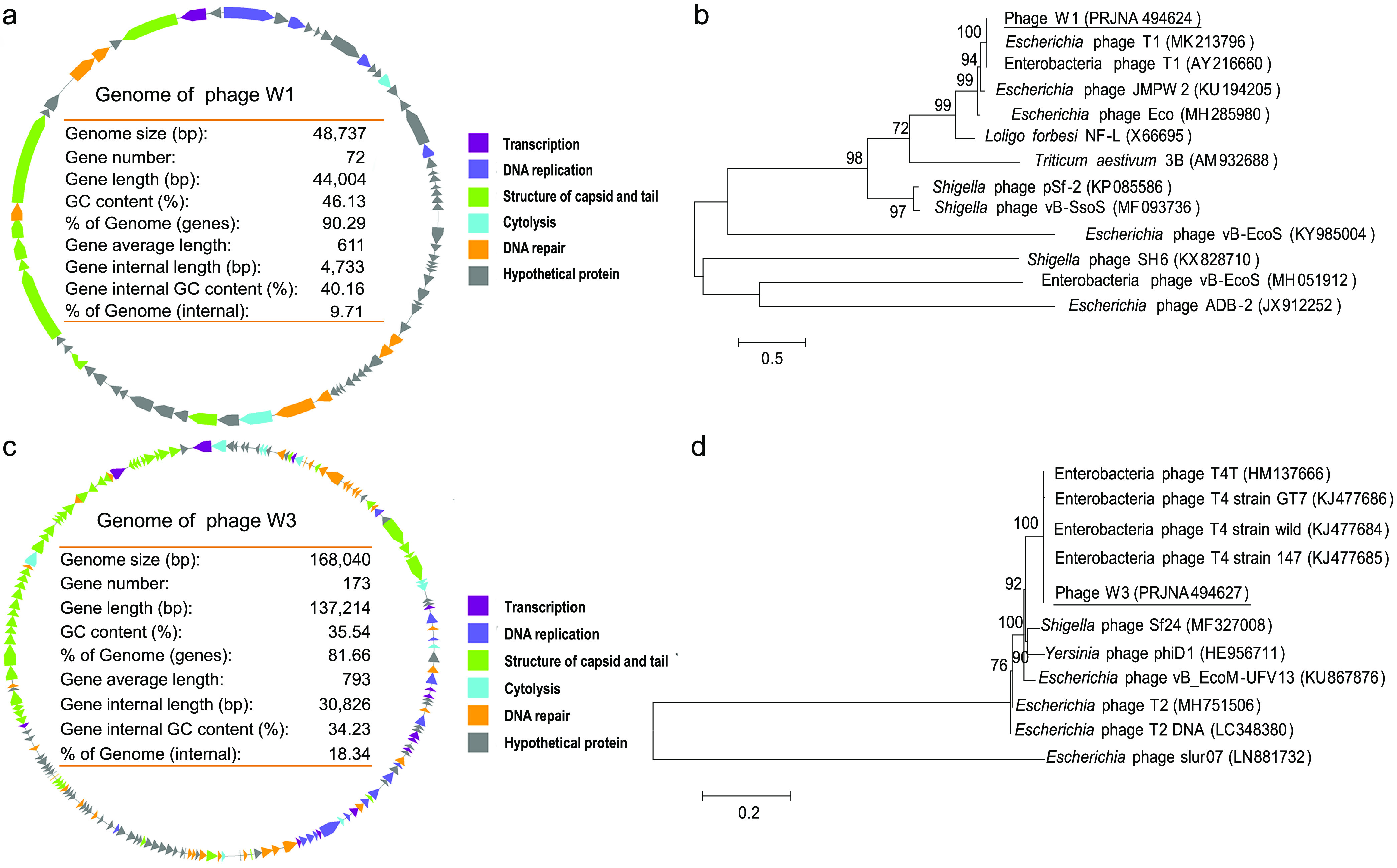
Genome characteristics and phylogenetic analysis of phages W1 and W3. Circular representation of the linear genome of phage W1 and its characteristics (a); phylogenetic tree of phage W1 based on the complete genome sequence (b); circular representation of the linear genome of phage W3 and its characteristics (c); and phylogenetic tree of phage W3 based on the complete genome sequence (d). (b and d) Confidence values above 50% obtained from 1,000-replicate bootstraps are indicated at branch nodes. The scale bars indicate the numbers of base substitutions per site.

Two phages, W1 and W3, were isolated from mouse fecal samples and selected for further testing due to their high lysis ability against strain MG1655. Their plaques are shown in Fig. S1a in the supplemental material. Phage W1 had an icosahedral head (65 ± 2 nm in diameter) and a flexible tail with a length of approximately 100 ± 20 nm under transmission electron microscopy (TEM) (Fig. S1b). Compared with phage W1, phage W3 was larger, with a 100- ± 10-nm-wide icosahedral head and 150- ± 10-nm-long tail composed of the tail tube, base plate, short spines, and long tail fibers (Fig. S1c). Phage W1 contained a double-stranded DNA, and its complete genome consisted of 48,737 nucleotides with an average GC content of 46.13%. Gene annotation analysis showed that the phage contained 72 genes, and 8 genes encoded structural proteins of the capsid and tail (Fig. 1a). The genome of phage W1 showed the highest similarity to Escherichia phage T1 (GenBank accession numbers MK213796 and AY216660), with 99.9% identity on the basis of the 100% query coverage, indicating that it is an Enterobacteria phage T1-like virus (Fig. 1b). The double-stranded DNA genome of phage W3 was 168,040 bp, with an average GC content of 35.54%, and it encoded 173 proteins, including structural proteins of the capsid and tail, as well as cytolysis, DNA repair, transcription, and nucleic acid metabolism enzymes (Fig. 1c). The complete genome of phage W3 showed the highest similarity to Escherichia phages T4 (GenBank accession numbers of KJ477685, KJ477684, and KJ477686), with 99.8% identity on the basis of the 100% query coverage, indicating that the phage is an Enterobacteria phage T4 -like virus (Fig. 1d). In addition, we also obtained other phages, including T5- and T7-like viruses, although they were not used in this work due to their low lytic ability, and the appearance of these well-known phages in the mouse gut suggests that they are ubiquitous.

### Host range of phages.

**FIG 2:**
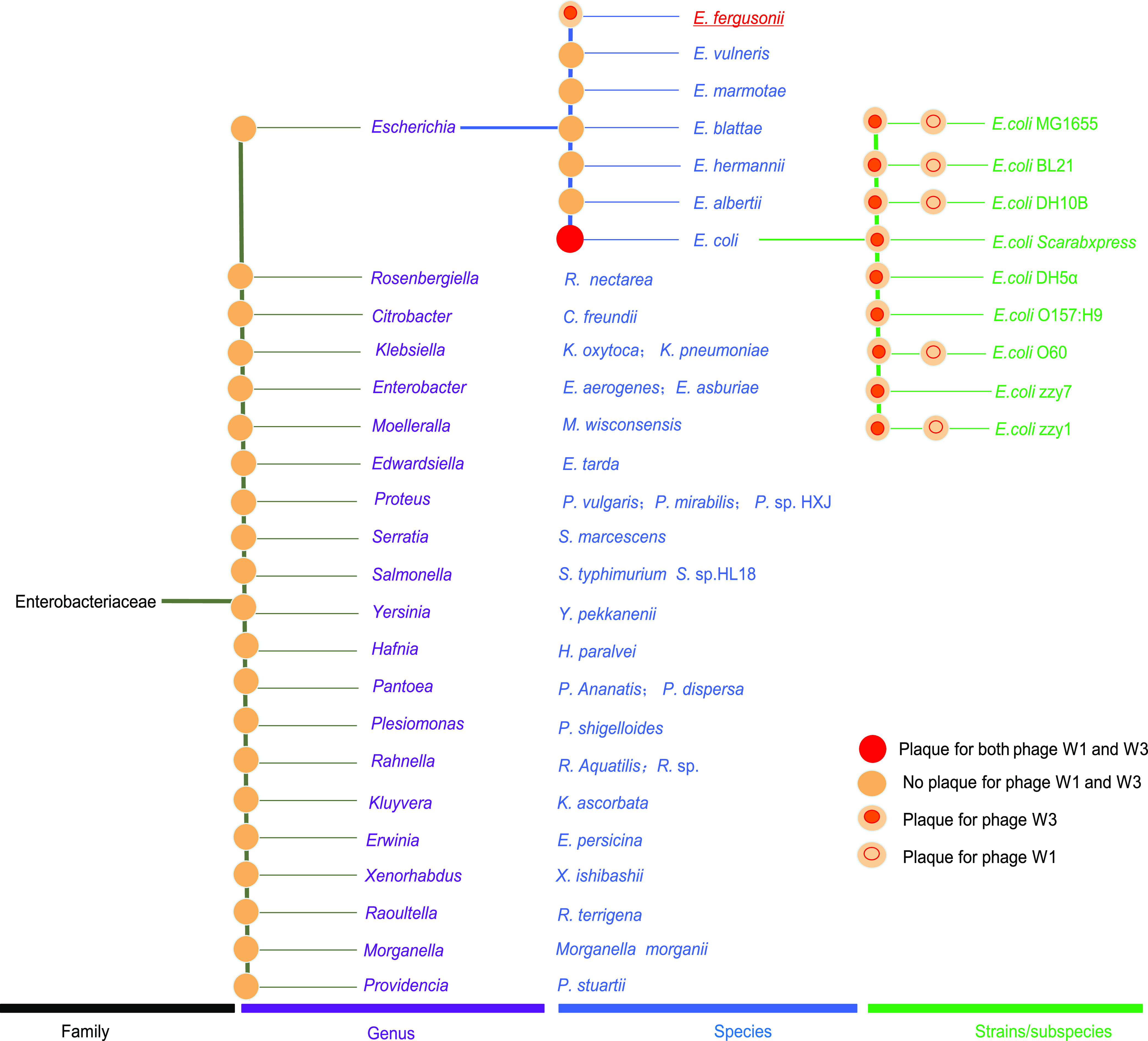
Host range analysis of phages W1 and W3.

Forty-five strains belonging to 21 genera were collected (Table S1) for host-specific analysis of phages W1 and W3. No plaque appeared on LB plates in spot tests, which indicated that neither phage infected these strains affiliated with 20 genera of *Enterobacteriaceae* (Fig. 2). Thus, the phages possessed host specificity at the genus level. At the species level of the genus Escherichia, phage W1 was not infectious to all six strains tested, while phage W3 could lyse Escherichia fergusonii, an emerging species within the genus Escherichia proposed by Farmer et al. (27), thus showing a wider host range than phage W1. In addition, phage W3 lysed all strains of E. coli, including different serotypes and natural intestinal strain representatives. Interestingly, phage W1 was not able to infect E. coli strains DH5α, Scarabxpress, O157:H7, and zzy7. Therefore, the phages could distinguish the different genera of the family *Enterobacteriaceae* and species of the genus Escherichia (except E. fergusonii), although they showed little host specificity at the strain level (28).

### Transformation of pGFPuv and stability analysis.

**FIG 3:**
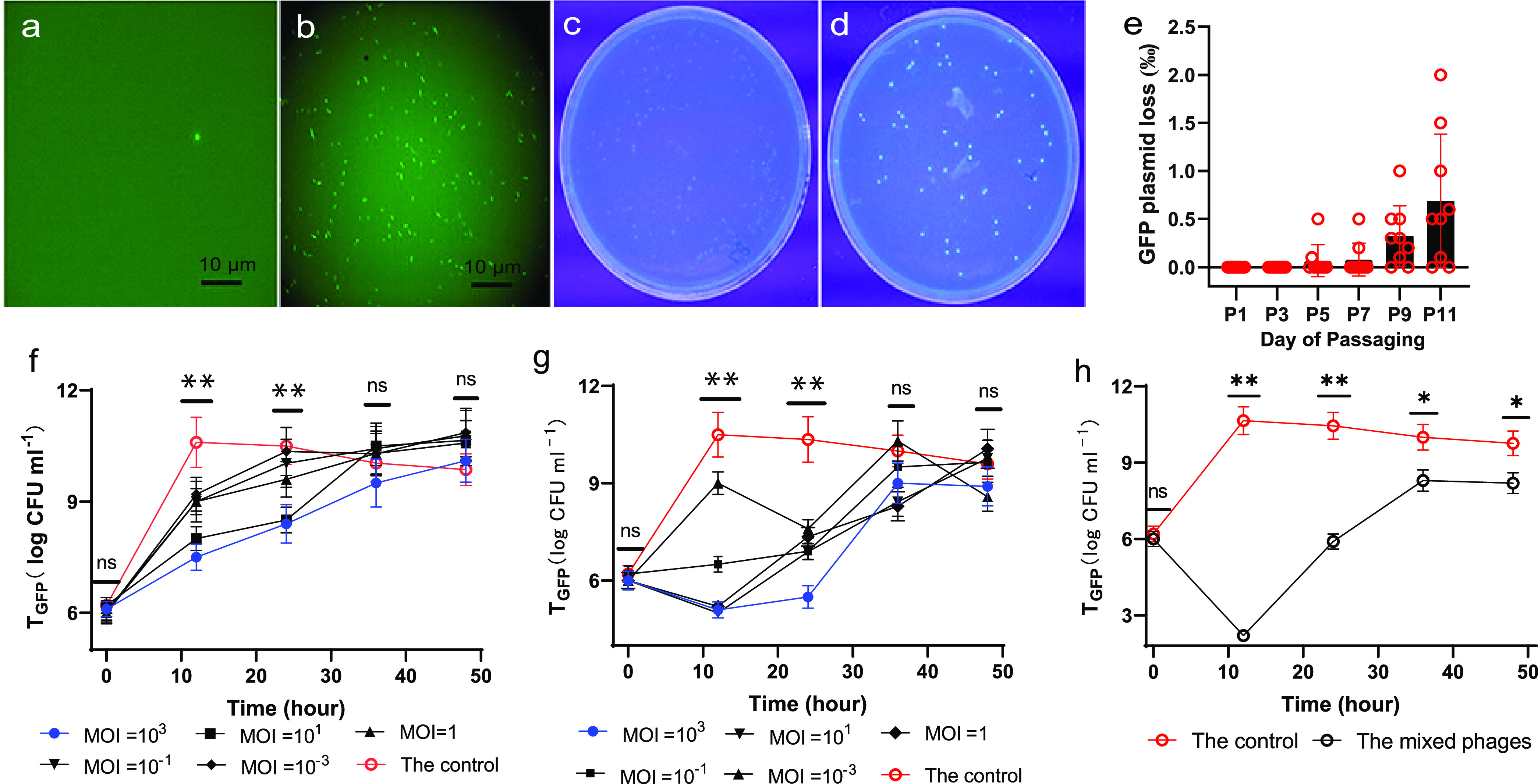
The pGFPuv transformation and phage infection tests *in vitro*. E. coli MG1655 (a) and T_GFP_ (b) under fluorescence microscopy; colonies of E. coli MG1655 (c) and T_GFP_ (d) on an LB plate when exposed to UV light; variations in pGFPuv loss during 11-day propagation (e); variations in T_GFP_ quantities with time during infection with phage W1 (f) and phage W3 (g) at various MOIs; and variations in T_GFP_ with time during infection with the mixed phages (h). The significance analyses in panels f and g show the differences between the plates with an MOI of 10^3^ and the control. ns, nonsignificant; *, *P* < 0.05; **, *P* < 0.01.

T_GFP_, the GFP-tagged transformant of E. coli MG1655, was prepared in order to differentiate the tested strain from the native E. coli strains in the mouse gut. Plasmid extracted from T_GFP_ was digested with the MluI enzyme, and electrophoresis showed that pGFPuv was successfully introduced into strain MG1655 (Fig. S2). Compared with the untagged strain, T_GFP_ with green fluorescence was easily observed under fluorescence microscopy (Fig. 3a and b). Moreover, it also exhibited blue and green fluorescence on LB plates when exposed to UV light (Fig. 3c and d), facilitating enumeration due to the green-fluorescent phenotype. Theoretically, plasmid loss for T_GFP_ occurs, since the plasmid is not integrated into the bacterial genome (29), and the enumeration of T_GFP_ requires plasmid stability. Following passages 1 to 11 in the absence of ampicillin, less than 1‰ average plasmid loss was obtained, indicating that the plasmid was relatively stable even after 11 days of propagation (Fig. 3e). Thus, the low plasmid loss supports the reliability of bacterial enumeration based on fluorescence labeling.

### Infection test *in vitro*.

Infection of T_GFP_ with phages W1 and W3 at various multiplicities of infection (MOIs) was monitored for 48 h, and the efficiencies of the different phages in reducing the titer of viable T_GFP_ varied. In the phage-free control, there was a rapid increase in the T_GFP_ concentration at 12 h. Compared with the control, infection with phage W1 resulted in a 0.2- to 3.5 log_10_ difference at 24 h at MOIs ranging from 10^−3^ to 10^3^. The infection test against W3 revealed that a high MOI was required for host lysis, and better reductions of viable bacteria were observed at both 12 h and 24 h with an MOI of 10^3^ (Fig. 3f and g). The maximum decline of variable bacteria reached 10^6^-fold less than that in the control at 12 h. However, it should be noted that neither phage was able to eliminate all bacteria, although W3 was more efficient than W1 in reducing viable bacteria.

During the infection of T_GFP_ with the mixed phages (MP [W1 and W3]) at an MOI of 10^3^, an 8.8-order of magnitude difference in the titer of viable bacteria was obtained at 12 h compared with that in the phage-free control, and the culture suspension in the test tube was clear. MP almost eliminated T_GFP_ at this point, although the culture finally recovered to a 10^8^ CFU mL^−1^ bacterial density at 48 h. Colonies with resistance against phage W1 were isolated from the cultures sampled at 48 h, and the phage predation induced the generation of resistant strains, although the bacterial resistance mechanism is still not clear (30).

### Bacterial colonization and removal *in vivo*.

**FIG 4:**
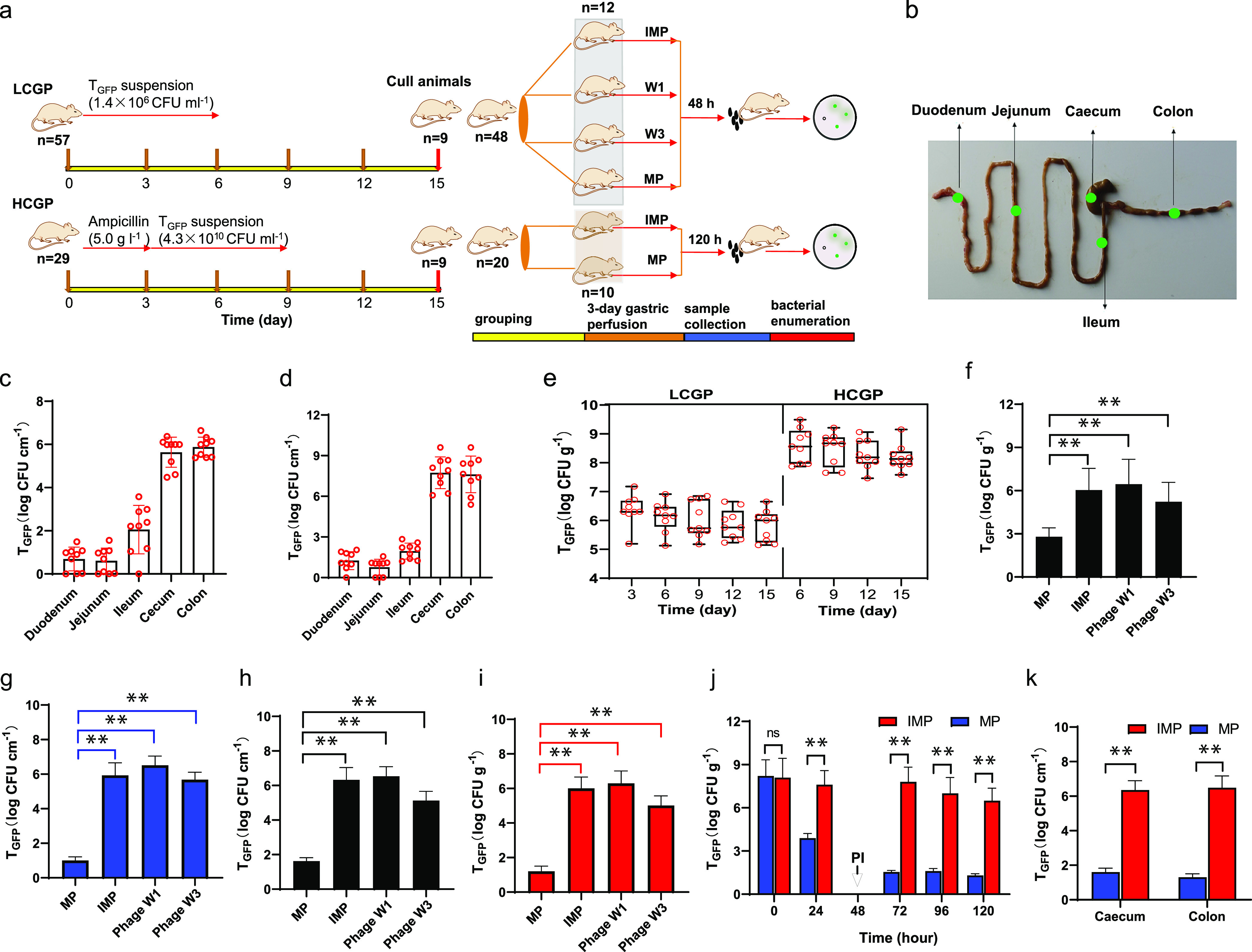
T_GFP_ removal test *in vivo*. Schematic representation of timing of different treatments for bacterial colonization and bacterial-removal test *in vivo* (a); sampling sites (b); T_GFP_ quantities in different intestinal sections in the LCGP (c) and HCGP (d) groups on day 15; T_GFP_ quantities in fecal samples in the LCGP and HCGP groups (e); T_GFP_ quantities in feces at 24 h after phage treatments in the LCGP group (f); T_GFP_ quantities in the cecum (g), colon (h), and feces (i) at 48 h after phage treatment in the LCGP group; variations in T_GFP_ quantities with time after two MP gastric perfusions in the HCGP group (PI, phage infusion) (j); and T_GFP_ quantities in the cecum and colon at 120 h after two MP treatments in the HCGP group (k). ns, nonsignificant; *, *P* < 0.05; **, *P* < 0.01.

Schematic representations of the timing of different treatments for bacterial colonization and removal tests and sampling sites in the mouse gut are shown in Fig. 4a and b. In order to investigate the removal of bacteria at different concentrations by phages, both low and high concentrations of T_GFP_ were administered to mice for bacterial colonization in the mouse gut. A 6-day low-concentration gastric perfusion (LCGP) with T_GFP_ suspension (10^6^ CFU mL^−1^) led to bacterial colonization in the mouse intestine. The quantity of T_GFP_ varied greatly in different intestinal sections. The cecum and colon were major sites of T_GFP_ colonization, and they harbored 9.0 × 10^6^ CFU cm^−1^ and 1.4 × 10^7^ CFU cm^−1^ of T_GFP_, respectively, on day 15 (Fig. 4c). After 3 days of pretreatment with ampicillin (31), a high-concentration gastric perfusion (HCGP) with 2.3 × 10^10^ CFU mL^−1^ of bacterial suspension resulted in the appearance of more T_GFP_ in the mouse gut, and on day 9, the bacteria in the cecum and colon reached up to 0.9 × 10^8^ CFU cm^−1^ and 1.38 × 10^8^ CFU cm^−1^, respectively (Fig. 4d). Variations of the amounts of T_GFP_ in feces are shown in Fig. 4e, and the viable bacteria in the LCGP and HCGP groups reached 0.60 × 10^6^ CFU g^−1^ and 0.62 × 10^8^ CFU g^−1^, respectively, on day 15, indicating stable colonization of T_GFP_ in the mouse gut. The cecum, colon, and feces were suitable to evaluate the bacterial-removal effect of phages on T_GFP_ in the mouse gut in the following studies, considering that they harbored more T_GFP_ than the other sites (23).

Subsequently, the results of the bacterial-removal test showed that bacteria in the mouse gut were almost eliminated by MP in the LCGP group. After gastric perfusion of MP, the T_GFP_ quantity in feces was 6.16 × 10^2^ CFU g^−1^ at 24 h (Fig. 4f), and at 48 h, T_GFP_ was undetectable in the cecum, colon, and feces (Fig. 4g to i). However, in the control mice treated with inactivated mixed phages (IMP) by gavage, bacteria in the cecum, colon, and feces remained at 6 orders of magnitude in CFU cm^−1^ or CFU g^−1^. In addition, phage W3 showed better bacterial removal than phage W1, although neither of them could eliminate T_GFP_. Therefore, only MP was selected for bacterial removal in subsequent experiments. The stability of pGFPuv was checked previously *in vitro*, but the fitness of T_GFP_ under phage predation in the mouse gut has not been revealed. T_GFP_ quantities in the phage W1 and W3 groups had no significant difference from the quantity in the IMP group (Fig. 4g to i), indicating that the fluorescence plasmid in E. coli was still stable even under the phage predation.

In the HCGP group, after the first phage infusion, T_GFP_ in feces still reached up to 0.77 × 10^4^ CFU g^−1^ at 24 h. The second infusion was conducted at 48 h. At 72 h, the bacteria in feces decreased to 0.35 × 10^2^ CFU g^−1^ and then stayed stable until 120 h (Fig. 4j). Simultaneously, T_GFP_ quantities in the cecum and colon were 0.40 × 10^2^ CFU cm^−1^ and 0.22 × 10^2^ CFU cm^−1^, respectively (Fig. 4k), and T_GFP_ was almost undetectable by the plate colony-counting method. Thus, T_GFP_ in the HCGP group also decreased to a 10^2^ CFU cm^−1^ level after two gastric perfusions of MP.

### Impact of MP on the bacterial community.

**FIG 5:**
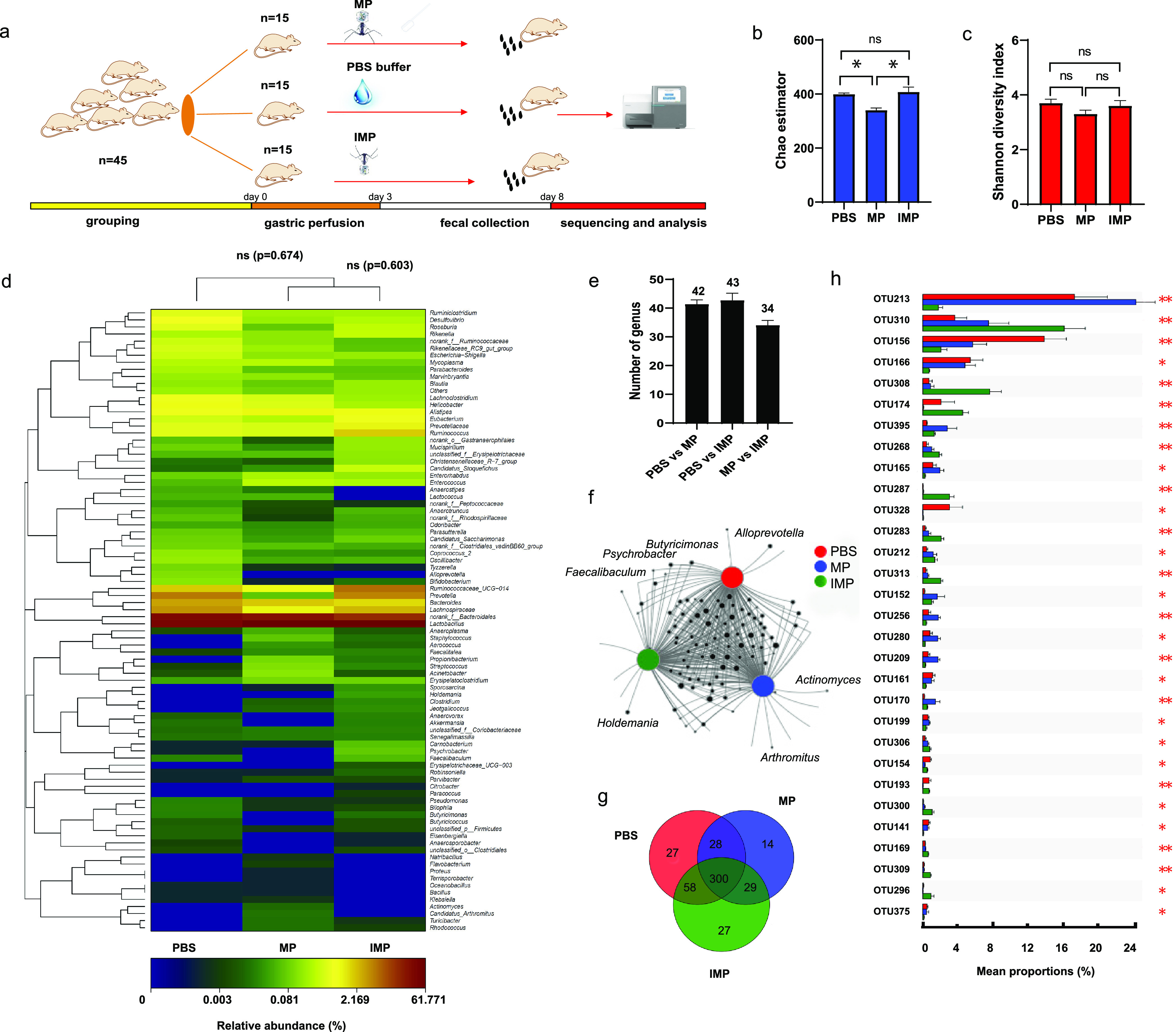
Impact of MP on the microbial community structure. Schematic illustration of the experimental design (a); the Chao1 index (b) and Shannon index (c) values of the samples on day 8 in the PBS, IMP, and MP groups; heatmap illustrating the changes in relative abundance (RA) at the genus level, with color scale below indicating the magnitude of RA (d); numbers of genera with significant RA differences among the MP, IMP, and PBS groups (e); network analysis of the cooccurrence of bacteria at the genus level (f); Venn diagram illustrating the OTU distribution among the MP, IMP, and PBS groups (g); and one-way ANOVA bar plot illustrating the OTUs with significant RA differences among the top 70 OTUs (h). ns, nonsignificant; *, *P* < 0.05; **, *P* < 0.01.

The design of experiments focused on the impact of MP on the bacterial community is shown in Fig. 5a. The orders of magnitude of native E. coli and its phages in mouse feces were at the 10^6^ and 10^2^ level, respectively, and the fecal samples on day 8 were used for sequencing since the quantities of both E. coli and phages in the MP group became stable after day 7 (Fig. S3). After 16S rRNA gene sequencing, trimmed sequences with an average sequence length of 398 bp were obtained, and statistical analysis of fecal samples, including the identified operational taxonomic units (OTUs) and alpha diversity indexes, are shown in Table S2. Good’s coverage indicated that 99.76 to 99.81% of the species in the samples were recovered at a cutoff of 97% sequence similarity. The Chao1 indexes of the phosphate-buffered saline (PBS), IMP, and MP groups were 399.2, 407.6, and 340.2, showing that the bacterial richness was significantly different after the phage treatments (Fig. 5b). The Shannon indexes of the three groups showed that phage treatment had no significant impact on bacterial diversity in the mouse gut (Fig. 5c). Dissimilarity comparison tests between the different groups at the genus level (Fig. 5d) also indicated that the addition of MP did not change the main microbial community structure (*P* > 0.05) (32).

We analyzed the changes in the relative abundances (RA) of bacteria in different treatments at the genus level because of the limited accuracy of molecular identification based on 16S rRNA gene sequencing at the species level. There were 42 and 43 genera with significant RA differences in the MP and IMP groups (Fig. 5e) compared with the PBS group, indicating that the disturbance caused by MP, defined as the number of bacterial genera with a significant RA change, was not significantly different from that in the IMP treatment (*P* > 0.05). In addition, the RAs of 34 genera changed significantly between the MP and IMP groups. Cooccurrence network analysis showed that the three groups shared the great majority of the species. Twelve genera, including Faecalibaculum, Butyricimonas, and Psychrobacter, appeared in both the PBS and IMP groups but were lost in the MP group (Fig. 5f). According to the previous host range analysis of E. coli phages, the disappearance of these bacteria was obviously not caused by an off-target effect. Therefore, treatment by live phages resulted in remodeling of the bacterial community in the mouse gut. However, whether in the IMP or MP group, the core top 30 genera (total RA of >98.5%) did not disappear (Fig. S4), and a similar conclusion could be drawn based on the OTU analysis. Among 483 OTUs in total, 58 OTUs present in the controls (IMP and PBS groups) were absent in the MP group (Fig. 5g). However, the core 70 OTUs accounting for the total RA of 93.9% were not lost (33), although the RAs of 30 OTUs showed significant differences (Fig. 5h). Therefore, it is feasible to edit a core member of the gut flora with native phages. The disappearance of noncore microorganisms or OTUs showed the reconstruction of the bacterial community structure. It is necessary to evaluate their interference during verification of the function of the target bacterium, and thus, further microbial supplementation experiments should be considered.

### Microbial interactions.

**FIG 6:**
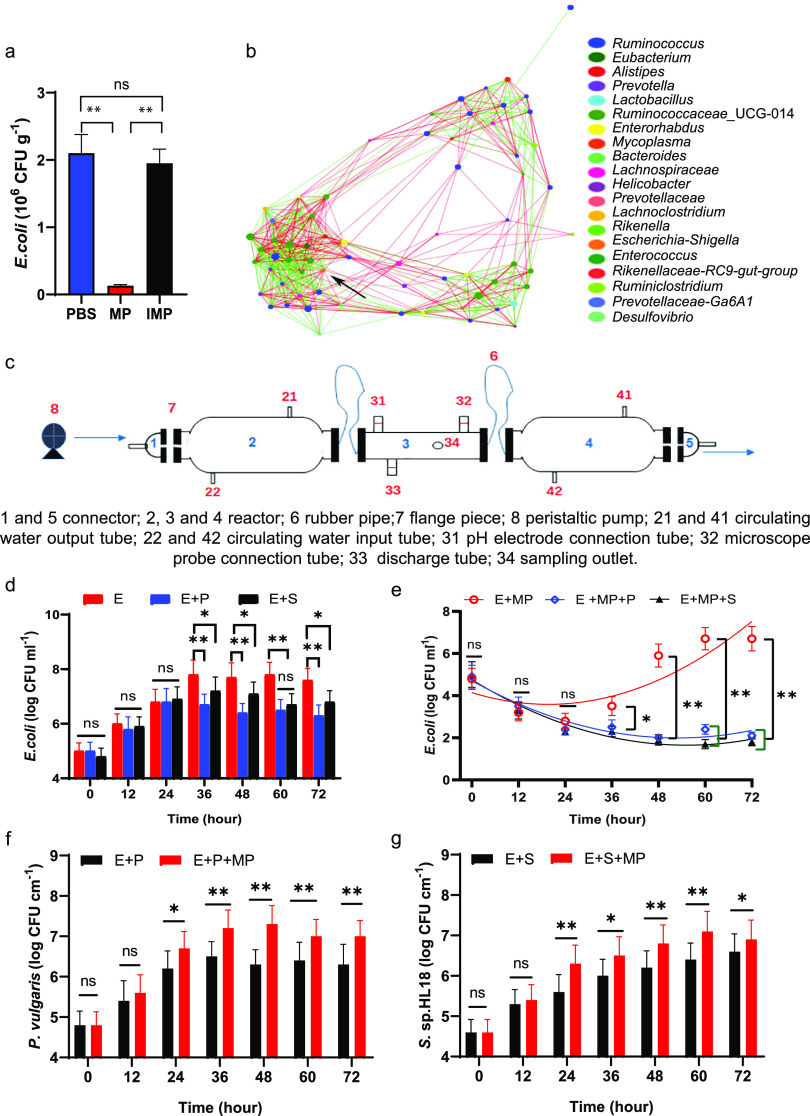
Enumeration of E. coli and correlation network analysis in the bacterial-removal and -coculture tests. (a) Quantities of E. coli in the MP, IMP, and PBS groups on day 8. (b) Correlation network analysis reveals the bacterial interactions in fecal samples. The sizes of the nodes show the abundances of OTUs (top 70), and the different colors indicate the corresponding taxonomic assignment at the genus level. The edge colors represent positive (red) and negative (green) correlations. The edge thicknesses indicate the correlation values, and the black arrow points to Escherichia. (c) Design of biomimetic reactors simulating the human intestinal tract. (d to g) Variations in the quantities of T_GFP_ (E) with time during coculture with Proteus vulgaris (P) and Salmonella sp. HL18 (S) (d); effect of MP on the quantities of T_GFP_ during coculture (e); and variations of the quantities of Proteus vulgaris (f) and Salmonella sp. HL18 (g) with time in visual biomimetic reactors during coculture. ns, nonsignificant; *, *P* < 0.05; **, *P* < 0.01.

The RAs of the Escherichia genus in the MP group (Fig. 5d) were significantly lower than those in the controls (*P* > 0.05), and enumeration results on day 8 showed that E. coli was reduced by approximately 1.4 orders of magnitude (Fig. 6a) in the MP group. However, neither the Escherichia genus nor E. coli was knocked out completely, because in addition to E. coli, the genus might contain species that are not sensitive to phages W1 and W3, and the MP we used targeted strain MG1655 rather than the entirety of strains of E. coli in the mouse gut. We isolated 1,000 colonies of E. coli from the fecal samples, and 406 colonies were insensitive to both phage W1 and W3 (Fig. S6). The Spearman correlation network analysis revealed that Escherichia was negatively correlated with 5 genera and positively correlated with 12 genera (*r* ≥ 0.6), indicating that it might affect the changes in RA of other genera through various cascade reactions (Fig. 6b). During coculture of E. coli and two common gut bacteria, Proteus vulgaris and Salmonella sp. HL18 (34, 35), in visual biomimetic reactors (Fig. 6c), the presence of these bacteria significantly reduced the E. coli population in both the E. coli plus P. vulgaris (E+P) and the E. coli plus Salmonella sp. HL18 (E+S) group (Fig. 6d). The addition of MP resulted in the knockdown of T_GFP_ during bacterial coculture, while this phenomenon was not observed in the control group (E+MP) (Fig. 6e). In addition, the phage predation against T_GFP_ resulted in increased P. vulgaris and Salmonella sp. HL18 titers compared with those in bacterial cocultures (Fig. 6f and g). These findings indicate that the interspecies competition improved phage control of the T_GFP_ population (36).

## DISCUSSION

### Feasibility of phage editing.

Microbial knockout is a novel issue in the microbiome field and is significantly different from gene knockout, which is used extensively for investigation of gene function. Generally, gene knockout is performed to remove one or several specific gene(s), while microbial knockout means that millions of microbial cells are eliminated. Thus, it is a huge challenge to achieve a complete elimination, especially given the high loads of bacteria. In both the LCGP and HCGP groups, we absolutely obtained a knockdown effect considering that the residual T_GFP_ population in feces was at a 10^2^ CFU g^−1^ level. The phage treatment significantly reduced the bacterial quantity but did not fully eliminate T_GFP_ in various colonization models, although the 10^2^ CFU g^−1^ bacterial load is almost undetectable by the plate colony-counting method due to the inevitable dilution of solid fecal samples. According to the growth dynamics of virulent phages, they rely on high concentrations of host bacteria to replicate, which explains why the MP are unable to eliminate low concentrations of T_GFP_. In addition, bacteria develop phage resistance during coevolution with phages. In this work, we isolated colonies resistant to phage W1. Therefore, considering the resistance against phages developed by bacteria, the limited contact between phages and host bacteria in the mouse gut, and other causes (24, 37), a strict bacterial knockout might be very difficult by means of phage treatment, and we should consider how to define microbial knockout within a broader range. As a whole, the ability of phages to reduce a target bacterium colonizing the mouse gut at concentrations of 10^6^ to 10^8^ CFU g^−1^ has satisfied the need for studying the bacterial function. Therefore, it is feasible to use phages as an editing tool for gut microbiota.

Normally, growing animals live in a poor-hygiene environment, and their intestines are open systems. They always have an opportunity to obtain bacteria from the environment, including the air, food, and water, even if the bacteria have already been eliminated in the gut. Therefore, it is unrealistic to expect to completely eliminate a specific microorganism in an unsterilized environment, and prevention of the continuous input of exogenous microorganisms is also an important issue during microbial editing, although generally speaking, the intake of microorganisms does not result in colonization in most cases due to the stability of gut microbiota.

### Antibiotic and intragastric treatment.

Strain MG1655, as an exogenous E. coli strain, does not normally colonize the mouse gut, and thus, ampicillin was administered to mice in the HCGP group prior to bacterial gavage to facilitate colonization. However, the preantibiotic treatment inevitably altered the structure of the normal gut microbiome (31). Also note that the bacterial titers achieved for E. coli colonization in this work were very high in the HCGP group, and this had a significant impact on both the composition of the underlying microbiota and phage dynamics. The goal of the study is to evaluate the feasibility of using phages as a microbiome editing tool to investigate a bacterial function, and thus, obviously, the ampicillin and intragastric treatments are no longer suitable for studying the functional aspects of the intestinal physiology. According to our measurements, the quantity of E. coli in the gut of normal mice is at a level of 10^5^ to 10^6^ CFU g^−1^, and neither antibiotic nor intragastric treatment is required during the actual bacterial editing, since the target bacteria are naturally present in the gut. Thus, ampicillin treatment in this experiment was only to facilitate the bacterial colonization, because the establishment of a model containing a high titer of bacteria was necessary to verify the capacity of phages in removing high-load bacteria in microecosystems, although a bacterial load as high as 10^8^ CFU g^−1^ is rare in the mouse gut.

### Off-target analysis.

The possibility of off-target effect should be evaluated during microbial editing by phages. Host specificity analysis of the phages showed that although the two phages possessed strict host specificity, phage W3 might infect E. fergusonii at the species level. E. fergusonii, formerly known as enteric group 10, is an infrequently occurring but emerging animal and human pathogen (38), and it can be differentiated from E. coli by sorbitol and lactose fermentation negativity but adonitol, amygdalin, and cellobiose fermentation positivity. E. coli and E. fergusonii are the closest relatives of one each other under the genus Escherichia, and E. fergusonii isolates possess genotypic and phenotypic features found in known pathotypes of E. coli, which leads to the following argument: is E. fergusonii in fact another E. coli? This question remains unanswered (39), and thus, we retested the bacterial classification based on the 16S rRNA gene sequence. A 1,421-bp gene sequence of E. fergusonii was determined, and the sequence comparison showed that the strain (GenBank accession number MK168572.1) shared the highest similarity (100%) with E. coli (accession number CP020516.1). According to the current opinion from molecular identification based on the 16S rRNA gene, this strain is an E. coli in nature by phylogenetic analysis (Fig. S5). An off-target effect did not occur at the species level if the taxonomic deviation is considered. However, this does not mean that an off-target effect will never occur during future microbial editing, because we only tested the reported strains under the Escherichia genus. In addition, phage host jumps have already been described in the mouse microbiota (40).

Overall, the results presented herein demonstrate that it is feasible to edit a specific bacterium by using its corresponding phages in the mouse gut. The native phages with host specificity can knock down T_GFP_ at a concentration of 10^6^ to 10^8^ CFU g^−1^ to a 10^2^ CFU g^−1^ level. Such an approach is undoubtedly of interest from the perspective of developing an informed gut engineering method. The use of phages for controlling pathogens was already conducted in the past. The administration of phages targeting Enterococcus faecalis was recently performed to control alcoholic hepatitis in mice, and the efficacy of phage predation against Vibrio cholerae, Clostridium difficile, and other pathogens was also investigated extensively (23, 25, 33, 41). These efforts focused on the use of phages for control of disease, rather than their use as a microbial editing tool in the gut or other microecosystems. In addition, it is known that a single nonnative phage may not be successful in phage therapy (26), and in this study, we proved that the combination of two native phages was more efficient than one native phage alone.

It should be noted that the removal of a specific bacterium should not be attributed entirely to the phages. In the mouse gut, T_GFP_ faces complicated ecological competition from various microorganisms. E. coli MG1655 is generally thought to be a noncoloniser. However, a transient colonization is enough for the phage treatment experiment. Thus, the rapid loss of T_GFP_ after phage perfusion is caused by the phages. In addition to phages, bacterial interactions also contribute to the reduction of T_GFP_ in the mouse gut, and this also explains why the resilience of T_GFP_ in *in vitro* phage treatment tests (Fig. 3h) was not apparent in the *in vivo* tests (Fig. S3a). Moreover, the changes in both the metabolomic and immune system caused by phage treatment were not evaluated in this work, considering that the phages isolated from mouse feces are native and the impact of MP on the bacterial community was not significant. However, these factors should be considered when assessing the function of a bacterial species in its host in future work.

## MATERIALS AND METHODS

### Microorganisms and chemicals.

All chemicals used were analytical grade. Forty-five bacteria, including E. coli strains, Escherichia species, and strains belonging to 20 different genera of *Enterobacteriaceae*, were purchased from the culture collections of China or provided by the Key Laboratory of Microbial Resources and Functional Molecules of Henan Province, China. Detailed information about these bacteria is listed in Table S1.

### Phage isolation and identification.

Phages W1 and W3 were isolated from mouse feces collected from the Animal Breeding Base in Henan Normal University, China. Fecal samples were seeded into 250-mL flasks containing 50 mL Luria-Bertani liquid medium (LBLM; 10 g L^−1^ tryptone, 5.0 g L^−1^ yeast extract, and 10 g L^−1^ NaCl) and incubated at 180 rpm for 5 h at 37°C. After filtration of the culture broth by gauze to remove large particles, the filtrate was centrifuged at 8,000 rpm for 10 min, and the supernatant was treated with a filter membrane (0.22 μm) to obtained filtrate 1. E. coli MG1655 was inoculated into 10 tubes containing 5 mL LBLM and incubated at 37°C and 150 rpm for 12 h. After inoculation of 1 mL of filtrate 1 into each tube, the culture was continued. Tubes in which the culture broth became clear were selected, and the broth was centrifuged at 8,000 rpm for 10 min. The supernatant was treated with a 0.22-μm filter membrane to obtain filtrate 2. Filtrate 2 (100 μL) and the suspension of E. coli MG1655 (100 μL) were mixed with 6 mL of LB soft agar (LB_SA_; LBLM supplemented with 10 g L^−1^ agar), and then the mixture was poured onto LB agar (LB_A_; LBLM supplemented with 15 g L^−1^ agar) plates. The culture was conducted at 37°C, and plaques appearing on the double-layer agar plate were used for phage propagation and identification.

Propagation of phages was conducted in LBLM by cocultivation with E. coli MG1655, and their titers were measured by the plaque assay method (42). After staining of phage particles with 2.0% aqueous uranyl acetate (pH 4.5 to 5.5) on a carbon-coated grid, phages were observed using transmission electron microscopy (TEM) (JEM-1400; JEOL Ltd., Japan) at an accelerating voltage of 80 kV. Phage DNA was extracted using a phage DNA extraction kit (Aidlab Biotech, China) (43). DNA sequencing was conducted using the Illumina HiSeq (PE250) platform at Hangzhou Lianchuan Biological Information Co., Ltd., China. The paired-end reads were assembled using SOAPdenovo version 2.04 (https://github.com/aquaskyline/SOAPdenovo2), and the potential open reading frames (ORFs) were predicted using GeneMarkS 4.6b (http://topaz.gatech.edu/GeneMark/). Possible tRNAs in the genome were determined using tRNAscan-SE (http://lowelab.ucsc.edu//tRNAscan-SE/). Comparisons of nucleic acid and predicted protein sequences with other known sequences were performed by BLAST analysis (http://blast.ncbi.nlm.nih.gov/Blast.cgi). Maps of the linear phage genomes were circularly represented using DNAMAN (version 6.0; Lynnon Biosoft). Neighbor-joining trees were drawn using MEGA 5.05 (44). The complete genome sequences of phages W1 and W3 were deposited in GenBank under accession numbers PRJNA494624 and PRJNA494627, respectively.

### Host range analysis of phages.

Strains were cultivated in LBLM for 24 h, and then bacterial lawns were prepared by pouring 3 mL of LB_SA_ containing 0.1 mL of overnight culture onto LB_A_. After solidification of the soft agar, the plates were spotted with a phage suspension (10 μL at 10^5^ to 10^6^ PFU mL^−1^) on triplicate plates. The plates were dried for 15 min at room temperature (25 ± 2°C) before incubation. After 20 h of incubation at 37°C, the effects of phages on bacterial lawns were observed and recorded. In the spot test, bacterial culture without phage was used as a negative control, and a positive result was defined as ≥20 plaques or full lysis on the plates.

### Fluorescent labeling of E. coli MG1655.

The plasmid pGFPuv, including a pUC origin of replication (ColE1-like), TEM β-lactamase as a selectable marker, and a cycle green fluorescent protein (GFP) reporter gene under the control of the Plac promoter (Clontech, CA, USA), was used to obtain a GFP-tagged transformant of E. coli MG1655 (T_GFP_). The plasmid was transformed into strain MG1655 by the heat shock method (45). Briefly, 100 μL of chemically competent cells and 100 ng of plasmid DNA were mixed and incubated on ice for 30 min; then, they were heat shocked in a water bath at 42°C for 90 s, allowed to recover for 1 h, and plated in the presence of 100 μg mL^−1^ of ampicillin. T_GFP_ was verified by PCR amplification after digestion with the MluI enzyme (TaKaRa Biotechnology Dalian Co., Ltd.) and fluorescence microscopy observation.

To evaluate the stability of the transformants in the laboratory, T_GFP_ was picked from plates and incubated in a 250-mL flask containing 50 mL of LBLM at 37°C and 120 rpm. After 24 h of cultivation, the cultures obtained were set as passage 1 (P1). Subsequently, 2 mL of culture broth from P1 was transferred to 50 mL of fresh LB medium every 24 h, and this procedure was repeated 10 times (46). To determine plasmid loss, cell cultures (2 mL) were collected at different passages, serially diluted, and plated on LB_A_. Colonies with green fluorescence under UV light were counted, and plasmid loss was expressed as the percentage of viable cells with abolished GFP fluorescence out of the total cells.

### Preparation of phage stocks.

Each phage suspension, prepared using sterile salt-magnesium buffer, was plated onto LB_A_ along with LB_SA_ containing 10^7^ CFU mL^−1^ of T_GFP_ overlay. After an overnight incubation at 37°C, salt-magnesium buffer was added to the plates, and the top soft agar slurry was harvested and centrifuged twice at 12,000 × *g* for 20 min to collect phage-rich supernatant (lysate). The lysate was added with an equal volume of chloroform and then filtered using a 0.22-μm filter to remove cells and debris. After titer measurement, the filtrate was serially diluted to obtain 10^3^ to 10^6^ PFU mL^−1^ phage stocks.

High-titer phage stocks were prepared from the lysates by liquid infection. For each phage, the low-titer lysate (1.0 mL at 10^6^ PFU mL^−1^) mixed with T_GFP_ (1.0 mL at 10^7^ CFU mL^−1^) was added to 200 mL of LBLM and incubated for 24 h at 37°C with aeration. The cultures were each treated with an equal volume of chloroform, and the lysates were harvested twice by centrifugation (12,000 × *g* for 20 min), as well as by filtration with a 0.22-μm filter. After titer measurement, the filtrate was serially diluted to obtain 10^7^ to 10^11^ PFU mL^−1^ phage stocks.

### Bacterial removal *in vitro*.

T_GFP_ was cultivated in LBLM for 24 h, and 100-μL amounts of cultures with a concentration of 4.0 × 10^7^ CFU mL^−1^ were transferred to test tubes containing 5 mL of LBLM. Then, phage suspensions (100 μL) of W1, W3, and mixed phages (W1 and W3 at a ratio of 1:1 [MP]) with different titers (10^4^ to 10^10^ PFU mL^−1^) were seeded in different tubes. The tubes were incubated at 37°C, and T_GFP_ was enumerated every 12 h by the plate colony-counting method. Considering the effect of lysis on enumeration of samples with high multiplicities of infection (MOIs), the bacterial cells were washed repeatedly by salt-magnesium buffer before plating to avoid an underestimation of viable cells. The reproducibility of the tests was confirmed in three independent continuous cultures. A phage-free culture containing only bacteria was used as a control to demonstrate the absence of phage contamination.

### Bacterial removal *in vivo*.

Ten-week-old male Kunming strain mice with an average body weight of 21 ± 2 g (Laboratory Animal Center of Henan Province, China) were used for removal tests *in vivo*. All of the mice were caged separately, maintained in a 12-h light/dark cycle, supplied with water and a standard diet (65% carbohydrate, 11% fat, and 24% protein), and housed at 20 to 25°C. The mouse experiments were not performed in a blinded manner, and the experimental groups were randomly allocated.

We first investigated the colonization of T_GFP_ in the mouse gut. Mice (*n* = 57) in the low-concentration gastric perfusion (LCGP) group were administered 200 μL of T_GFP_ suspension (1.4 × 10^6^ CFU mL^−1^) by gavage once per day for 6 consecutive days. In the high-concentration gastric perfusion (HCGP) group, a 3-day gastric perfusion with 5.0 g L^−1^ ampicillin (47) was administered to mice (*n* = 29) to facilitate bacterial colonization before they were administered 200 μL of T_GFP_ suspension (2.3 × 10^10^ CFU mL^−1^) by gavage once per day for 6 days. After the treatments, T_GFP_ in mouse feces was enumerated daily. Mice (*n* = 9) from the LCGP and HCGP groups were dissected on day 15, and T_GFP_ in different sections of the gut was collected and enumerated to validate T_GFP_ colonization.

Forty-eight mice from the LCGP group were divided into four groups (*n* = 12) and were administered 100 μL of MP (1.2 × 10^6^ PFU mL^−1^), phage W1 (1.1 × 10^6^ PFU mL^−1^), phage W3 (1.4 × 10^6^ PFU mL^−1^), or inactivated mixed phages (IMP) by gavage. As a control, IMP was prepared by sterilization of MP at 121°C and 0.1 to 0.15 MPa of steam pressure for 30 min in an autoclave (Hiclave HVE-50). After 3 days of gastric perfusion, T_GFP_ in mouse feces was enumerated every 24 h.

Twenty mice from the HCGP group were divided into two groups (*n* = 10) to quantitatively evaluate the removal efficiency of MP against the high titers of T_GFP_. The two groups were administered 100 μL of MP (1.8 × 10^9^ PFU mL^−1^) or IMP by gavage. After 3 days of gastric perfusion, T_GFP_ in mouse feces was enumerated every 24 h, and T_GFP_ in the cecum and colon was evaluated at 120 h.

### Phylogenetic analysis of *E. fergusonii*.

Genomic DNA of E. fergusonii (bacterial strain CICC24137) was extracted, and the 16S rRNA gene sequence was amplified using PCR with the primer pair 27F and 1492R (48). The purified PCR product was cloned into the vector pMD19-T and sequenced. Sequences of related taxa were obtained from the GenBank and EzTaxon-e databases. Phylogenetic analysis was performed using MEGA software version 5.05 after multiple alignment of data by DNAMAN. Evolutionary distances and clustering were constructed by the neighbor-joining method.

### Experimental design and 16S rRNA gene sequencing.

Forty-five Kunming mice were used to test the impact of phage treatment on the microbial community structure. Mice in the MP group were administered MP once per day for 3 days by oral gavage, and as controls, the PBS and IMP group were administered PBS buffer or IMP, respectively, by gavage. During the tests, changes in movement, appetite, dejecta, phages, and E. coli titers in fecal samples were recorded. E. coli was enumerated by using the CHROMagar E. coli chromogenic medium (CHROMagar, France) (49). Fecal pellets of mice (20 mg per mouse) from the three groups were collected, and samples obtained on day 8 were selected for sequencing and shaken sufficiently for 30 min in a 50-mL sterile centrifuge tube. Subsequently, both sterile gauze and 5-μm filter membranes were used to remove large particles, and the filter liquor was used for further genomic DNA extraction.

Bacterial genomic DNA in fecal samples was extracted using an Omega Bio-Tek soil DNA kit (Qiagen, Germany). After verification of the purity and concentration, PCR amplifications of the highly variable V3-V4 regions of the bacterial 16S rRNA gene were conducted based on the universal primer pair 338F (ACTCCTACGGGAGGCAGCAG) and 806R (GGACTACHVGGGTWTCTAAT).

The thermocycling procedure consisted of an initial denaturation step at 95°C for 2 min, followed by 25 cycles of 94°C for 30 s (denaturation), 55°C for 30 s (annealing), and 72°C for 30 s (extension) and then a final extension at 72°C for 5 min. Each reaction was conducted in a 20-μL reaction mixture containing 10 ng of template DNA, 5 μM each primer, 2.5 mM deoxynucleoside triphosphate mix, and 1 unit of FastPFU polymerase (TransGen Biotech, China). PCR cycling reactions were performed in a GeneAmp 9700 DNA thermocycler (ABI, USA), and the amplified products were visualized on agarose gel containing EB and purified with a DNA gel extraction kit (Axygen, Inc., USA).

Prior to sequencing, the DNA concentration of each PCR product was determined, and the amplicons from each PCR were pooled in equimolar ratios to reduce the biases of each individual reaction and then subjected to emulsion PCR to generate amplicon libraries (50). Deep sequencing was performed on the Illumina MiSeq platform at the Majorbio Bio-pharm Technology Co., Ltd. (Shanghai, China). Any sequence with more than two base mismatches was discarded by Seqcln software analysis. The low-quality sequences and redundant reads were further trimmed using mothur software. The “dist.seqs” command was performed to identify operational taxonomic units (OTUs) by 97% similarity. The obtained sequences were subjected to Megablast and searched against SILVA, aligning to the 16S small subunit rRNA sequence database (version 111), to acquire high taxonomic resolution. The rarefaction curves, Chao1 richness, and Shannon diversity index were determined by Mothur analysis (51).

### Bacterial-coculture experiments.

T_GFP_ was cocultivated with Proteus vulgaris and Salmonella sp. HS18 in visual biomimetic reactors that can simulate peristalsis of the human intestinal tract (52), and a monoculture of T_GFP_ was used as the control. In addition, MP (10 mL at 10^8^ PFU mL^−1^) was added to the reactors to validate the clearance effect on T_GFP_ during bacterial coculture. The medium used contained corn starch (20.0 g L^−1^), protein powder (20 g L^−1^), glucose (5.0 g L^−1^), Ox-gall salt (10.0 g L^−1^), Na_2_HPO_4_ (10.0 g L^−1^), KH_2_PO_4_ (10.0 g L^−1^), and NaCl (1.0 g L^−1^), and the initial pH value was 7.2 to 7.5. In the tests, the approximate inoculation quantities of the bacteria were 10^5^ CFU mL^−1^. The compression frequency of the peristaltic pump was 10 times per minute, and the compression range was 1 cm. The cocultures were carried out for 72 h. T_GFP_ was enumerated every 12 h by the plate colony-counting method, and the quantity of P. vulgaris or Salmonella sp. HS18 was calculated by subtracting the number of T_GFP_ from the total number of bacteria.

### Statistical methods.

Statistical analyses to identify significant differences were performed using SPSS software (version 19.0). Unless otherwise specified, all data are presented as the mean value ± standard error of the mean (SEM). When three or more means were compared for statistical significance, one- or two-way analysis of variance (ANOVA) was conducted with treatments as independent factors. When two groups of measurements were examined for statistical significance, the two-sided Student’s *t* test was conducted, and a *P* value of <0.05 was considered statistically significant. A dissimilarity test of fecal samples was performed in R based on the Bray-Curtis dissimilarity index using analysis of similarities. Cooccurrence and correlation network analysis were performed by using Networkx software, and only Spearman correlations with an *r* of >0.6 (*P < *0.05) were considered to indicate a valid interactive event.

### Ethics approval.

Animals were treated according to the guidelines of the Regulations for the Administration of Laboratory Animals (Decree No. 2 of the State Science and Technology Commission of the People’s Republic of China, 1988). The sampling procedure was validated by the Ethics Committee of the Henan Normal University.

### Data availability.

The data sets generated and analyzed during the current study, including genome sequences of phages W1 and W3 and 16S rRNA gene sequencing data, are available in the NCBI Sequence Read Archive (SRA) under accession numbers PRJNA494624, PRJNA494627, and PRJNA578836.
