# Supplementary material for: Editing of a Specific Strain of Escherichia coli in the Mouse Gut Using Native Phages
Source: Microbiol Spectr. 2022 Oct 27;10(6):e01804-22. doi: 10.1128/spectrum.01804-22 (PMC9770003; doi:10.1128/spectrum.01804-22)
Supplement: Supplemental file 1 — Tables S1 and S2 and Fig. S1 to S6. Download spectrum.01804-22-s0001.pdf, PDF file, 0.8 MB [file spectrum.01804-22-s0001.pdf]

Table S1 The detailed information about bacteria used in this work.

Strain	Source	Access number or isolates source
Citrobacter freundii (P)	CCTCC	CCTCC AB 206596
Citrobacter amalonaticus (P)	CCTCC	CCTCC AB 2010357
Klebsiella pneumoniae (P)	CCTCC	CCTCC AB 2012147
Klebsiella oxytoca (I)	LMRFM	Mouse feces
Enterobacter aerogenes (P)	CCTCC	CCTCC AB 2012953
Enterobacter asburiae (I)	LMRFM	Human skin
Enterobacter ludwigii (I)	LMRFM	Human skin
Moelleralla wisconsensis	LMRFM	Mouse feces
Edwardsiella tarda (P)	CCTCC	CCTCC AB 2013118
Proteus vulgaris (P)	CCTCC	CCTCC AB 91103
Proteus mirabilis (I)	LMRFM	Mouse feces
Proteus sp. (I)	LMRFM	Mouse feces
Serratia marcescens (P)	CGMCC	CGMCC 1.1857
Salmonella typhimurium (P)	CCTCC	CCTCC AB 2014173
Salmonella sp. HL18	LMRFM	Mouse feces
Yersinia pekkanenii (P)	CCTCC	CCTCC AB 2012518
Hafnia paralvei (P)	CCTCC	CCTCC S2014320
Pantoea ananatis (P)	CCTCC	CCTCC AB 204019
Pantoea dispersa（I）	LMRFM	Human skin
Plesiomonas shigelloides (P)	CCTCC	CCTCC AB 2010149
Rahnella aquatilis (P)	CCTCC	CCTCC AB 207693
Rahnella sp. (I)	LMRFM	Mouse feces
Kluyvera ascorbata (P)	CCTCC	CCTCC AB 205562
Erwinia persicina (P)	CCTCC	CCTCC S2014903
Rosenbergiella nectarea (P)	CCTCC	CCTCC S2013624
Xenorhabdus ishibashii (P)	CGMCC	CGMCC 1.9166
Raoultella terrigena (P)	CGMCC	CGMCC1.4008

Providencia stuartii (P)	CGMCC	CGMCC 1.1360
Phytobacter iazotrophicus (P)	CGMCC	CGMCC 1.5339
Morganella sp. (I)	LMRFM	Human skin
Escherichia marmotae (P)	CGMCC	CGMCC 1.12862
Escherichia vulneris (P)	CGMCC	CGMCC 1.3441
Escherichia hermannii (P)	CICC	CGMCC 1.3439
Escherichia albertii (P)	CGMCC	CICC 24149
Escherichia blattae (P)	CICC	CGMCC 1.3440
Escherichia fergusonii (P)	CGMCC	CICC 24137
E.coli K12/MG1655	LMRFM	
Escherichia coli K12/DH5α	LMRFM	
E.coli Scarabxpress (P)	LMRFM	
E.coli BL21	LMRFM	
E.coli K12/DH10B	LMRFM	
E.coli O157:H9	LMRFM	
E.coli O60	LMRFM	
E.coli zzy1 (I)	LMRFM	Mouse feces
E.coli zzy7 (I)	LMRFM	Mouse feces

Note:

CCTCC, China Center for Type Culture Collection; CGMCC, China General Microbiological Culture Collection Center; CICC, China Center of Industrial Culture Collection; LMRFM, Key Laboratory of Microbial Resources and Functional Molecules of Henan Province, China.

P, purchased from collection centers;

I, isolated from various environmental samples.

Table S2 Sequencing results and statistical analysis of fecal samples. PBS: samples from the PBS group; IMP: samples from the inactivated MP group; MP: samples from the MP group.

**Samples**	**Average Reads**	**Coverage (%)**	**Sobs**	**ACE estimator**	**Simpson's diversity**	**Shannoneven Index**
PBS	25300	99.80	366	399.39	0.10028	0.6321
IMP	27277	99.81	301	343.88	0.07398	0.5728
MP	26977	99.76	347	395.38	0.07917	0.6155

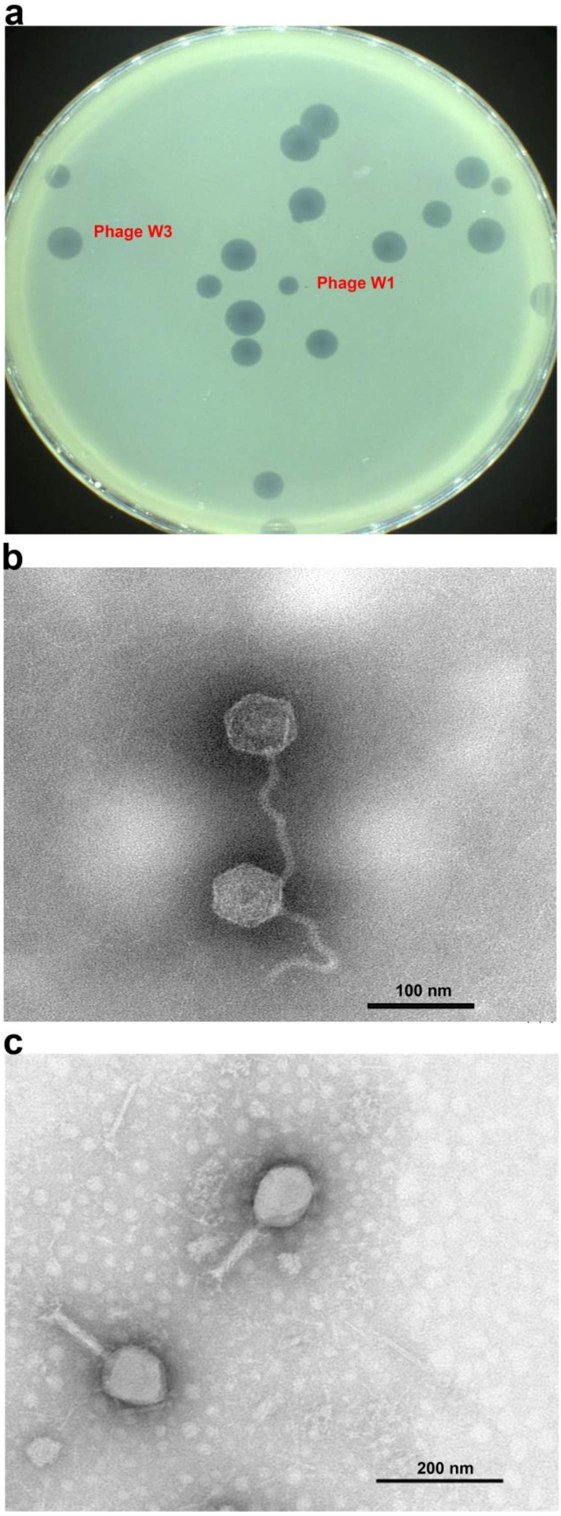

Fig. S1 Morphological features of phages W1 and W3. **(a)** their plaques. Morphological observation of phage W1 (**b**) and phage W3 (c) under transmission electron microscope.

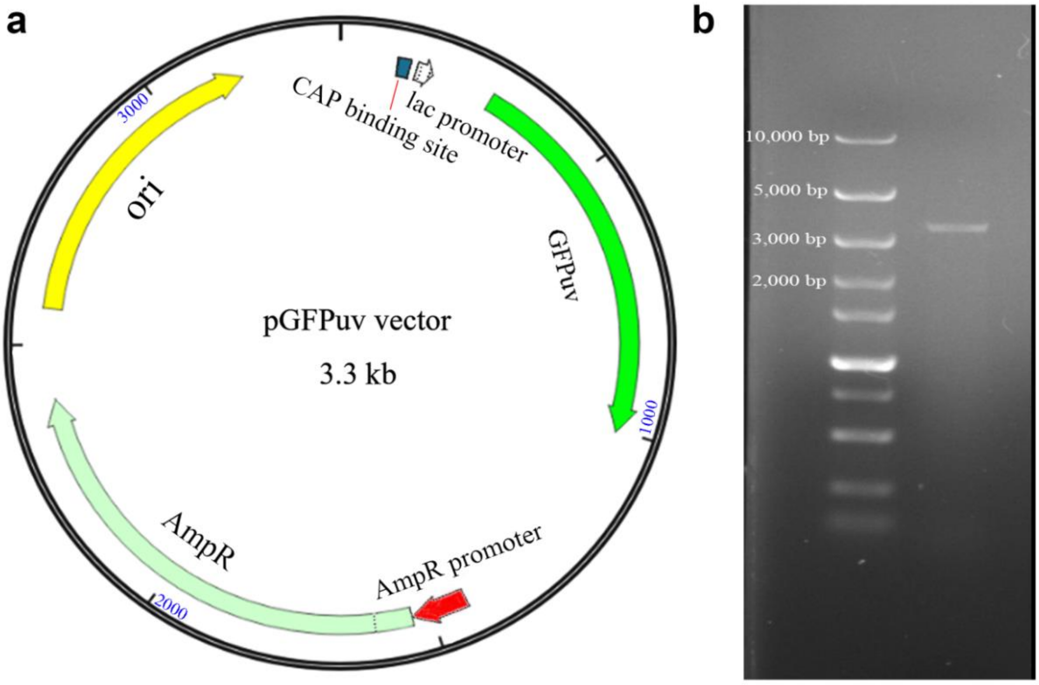

Fig.S2 The pGFPuv profile and verification of plasmid transformation. (**a)** the pGFPuv profile, and (**b)** verification of plasmid transformation by electrophoresis.

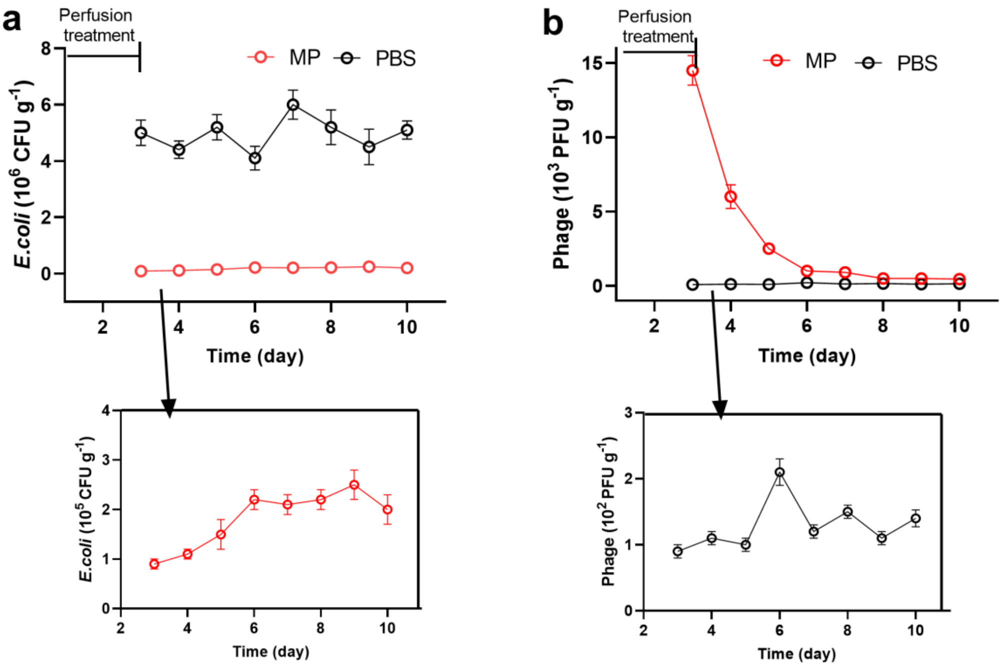

Fig. S3 Variations of titers of both *E. coli* and phages in fecal samples from the MP and PBS group. (**a)** *E. coli*, and (**b)** phages of *E. coli*. *E. coli* MG1655 was co-cultivation with phages to measure the titer of phages.

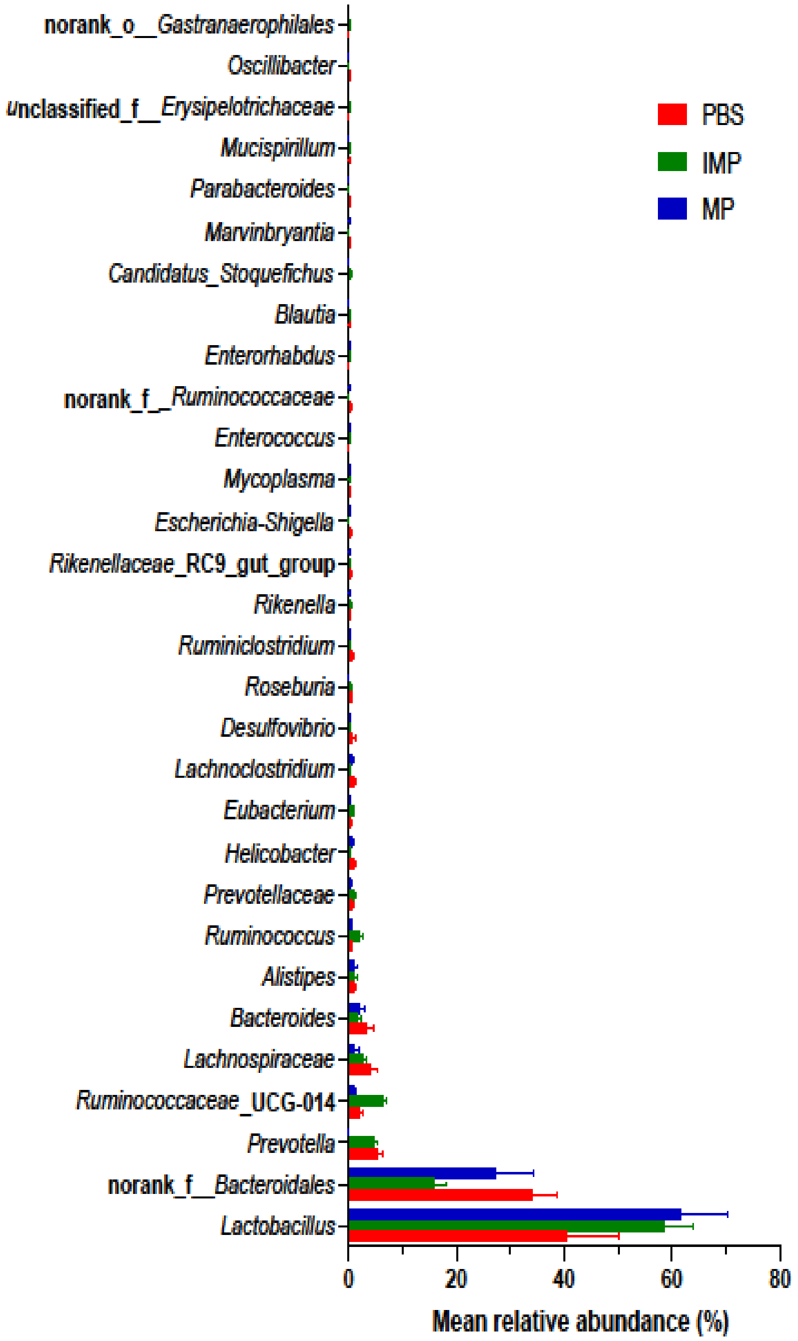

Fig. S4 Mean relative abundances of the top 30 genera in the IMP, MP and PBS group on day 8.

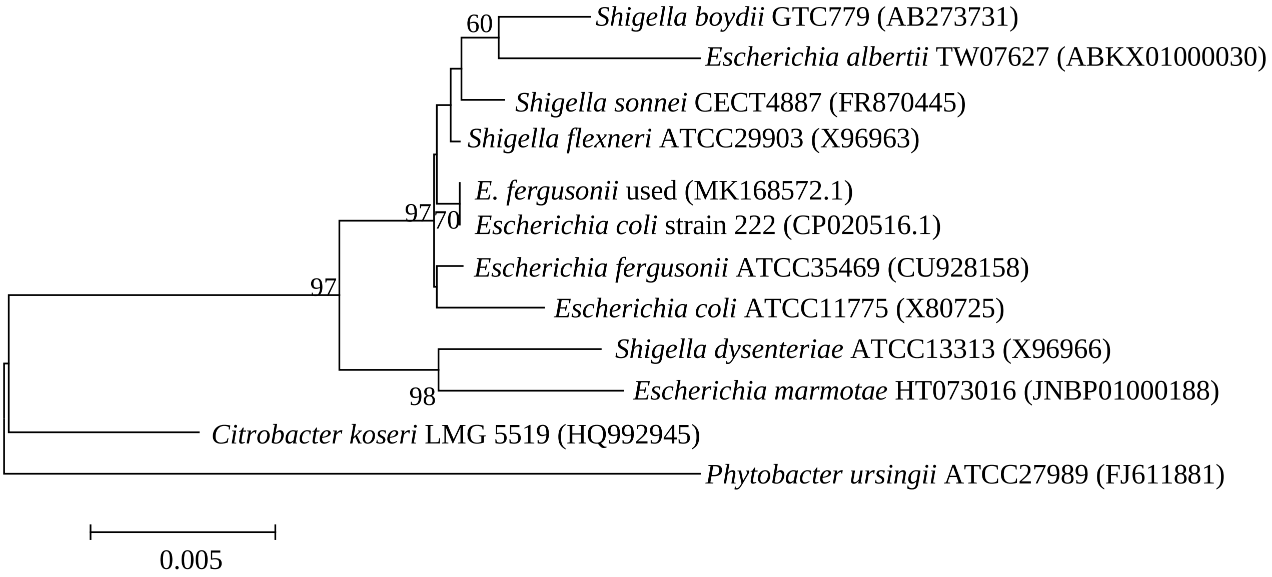

Fig. S5 Phylogenetic analysis of *E. fergusonii* used based on 16S rRNA gene sequence. Confidence values above 50% obtained from 1000-replicate bootstraps are indicated at branch nodes. The scale bar indicates the number of base substitutions per site.

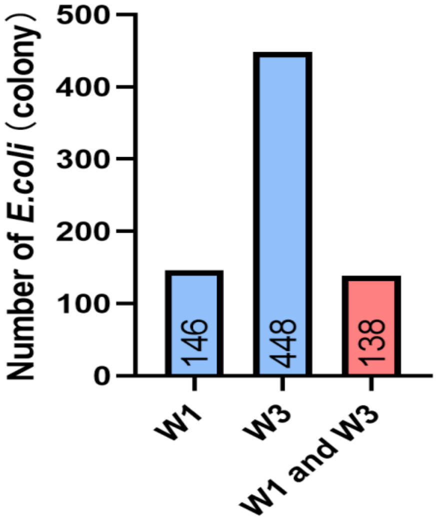

Fig. S6 Number of colonies of *E. coli* which are sensitive to phage W1, W3, and both W1 and W3. Totally 1000 colonies isolated from the fecal samples were analyzed.
